# Magnetic Nanoparticles as Intraocular Drug Delivery System to Target Retinal Pigmented Epithelium (RPE)

**DOI:** 10.3390/ijms15011590

**Published:** 2014-01-22

**Authors:** Martina Giannaccini, Marianna Giannini, M. Pilar Calatayud, Gerardo F. Goya, Alfred Cuschieri, Luciana Dente, Vittoria Raffa

**Affiliations:** 1 Institute of Life Science, Scuola Superiore Sant’Anna, Piazza Martiri della Libertà 33, Pisa 56127, Italy; E-Mails: mailto:m.giannini@sssup.it (M.G.); a.cuschieri@sssup.it (A.C.); 2 Instituto de Nanociencia de Aragon & Condensed Matter Physics Department, Universidad de Zaragoza, Mariano Esquillor edif. I+D, Zaragoza 50018, Spain; E-Mails: pilarcs@unizar.es (M.P.C.); goya@unizar.es (G.F.G.); 3 Department of Biology, Università di Pisa, S.S. 12 Abetone e Brennero 4, Pisa 56127, Italy; E-Mail: ldente@biologia.unipi.it (L.D.)

**Keywords:** magnetic nanoparticle (MNP), intraocular delivery, retinal pigmented epithelium (RPE), *Xenopus laevis*

## Abstract

One of the most challenging efforts in drug delivery is the targeting of the eye. The eye structure and barriers render this organ poorly permeable to drugs. Quite recently the entrance of nanoscience in ocular drug delivery has improved the penetration and half-life of drugs, especially in the anterior eye chamber, while targeting the posterior chamber is still an open issue. The retina and the retinal pigment epithelium/choroid tissues, located in the posterior eye chamber, are responsible for the majority of blindness both in childhood and adulthood. In the present study, we used magnetic nanoparticles (MNPs) as a nanotool for ocular drug delivery that is capable of specific localization in the retinal pigmented epithelium (RPE) layer. We demonstrate that, following intraocular injection in *Xenopus* embryos, MNPs localize specifically in RPE where they are retained for several days. The specificity of the localization did not depend on particle size and surface properties of the MNPs used in this work. Moreover, through similar experiments in zebrafish, we demonstrated that the targeting of RPE by the nanoparticles is not specific for the *Xenopus* species.

## 1. Introduction

Disorders of the retina and retinal pigmented epithelium (RPE)/choroid tissues, located in the posterior eye chamber, are responsible for the majority of blindness both in childhood and adulthood. The most prevalent posterior segment diseases include age related macular degeneration (AMD), diabetic retinopathy, and retinal degenerative disorders such as retinitis pigmentosa. The treatment with neurotrophic or anti-angiogenic factors, whilst not curative, may halt the disease process and it is thus a promising therapeutic approach [1]. Drug therapy is compounded by the limit in drug delivery to RPE resulting from the ocular anatomy and physiology. The cornea/sclera constitutes a static barrier severely limiting ocular bioavailability of drugs to ineffective levels after systemic administration. Hence, both routes of administration, surface instillation and intravenous delivery are in practice largely ineffective. Subconjunctival delivery is more invasive and still suffers from the same limitations in terms of access to the posterior eye chamber [2]. Intravitreal (IVT) and subretinal injections are generally considered as the most effective ways of delivering material to the back of the eye. In particular, subretinal injection seems to be the only effective option to target RPE. Unfortunately, subretinal injections are very invasive with reduced patient compliance compared to IVT injections [3]. IVT injection involves injection of drug in solution directly into the vitreous which is far from optimal for two reasons. In the first instance, the initial high drug concentration can provoke short term complications such as retinal detachment, endophthalmitis and intravitreal hemorrhages [4]. Secondly, the presence of vitreous humor is associated with a very short retention time and lack of tissue specificity (the vitreous humor surrounds different intraocular tissues). Therefore, long-term delivery of biologically active molecules to the RPE is problematic and remains a challenge. The use of various polymeric nanoparticles (NPs) to increase the drug bioavailability and improve the delivery kinetics has been investigated, as nanocarriers have been widely exploited in nanomedicine to provide sustained release of molecules at the desired sites [5,6]. Sustained eye prolonged drug release (over one month) was demonstrated by using polystyrene nanospheres containing a fluorescein derivative [7]. Interestingly, in one study 2 μm NPs were observed in the vitreous cavity and trabecular meshwork, and 200 nm NPs were reported to also be located in the retina and RPE [7]. In general, IVT injection of drugs, including nanoformulations, is not optimal for specific delivery to RPE. In order to gain specificity towards RPE, NP functionalization with ligands of various transporter/receptors (e.g., P-gp, MRP) has been proposed but not yet demonstrated [5]. The only studies demonstrating specific localization in RPE following IVT injection concern the use of naked polylactide (PLA) NPs and human albumin (HSA) NPs in normal rat eyes [8,9]. Specifically, the kinetics of intraocular tissue/cellular localization after a single IVT injection of PLA NPs revealed specific PLA NP *trans*-retinal movement, with a final localization in RPE cells (four months after the injection) [8].

The use of magnetic nanoparticles (MNPs) engineered for biomedical applications has grown significantly during the past two decades. This class of NPs include metallic, bimetallic, and superparamagnetic iron oxide nanoparticles (SPIONs) [10]. MNPs form a powerful drug delivery system because their reactive surface can be functionalized with biocompatible coatings, bioactive molecules or targeting-moieties to increase their specificity toward cellular targets and prevent interaction with healthy tissues [11–15]. In addition to their established role as molecular carriers, MNPs have two other advantages. They can be controlled by noncontact forces [16] and tracked by magnetic resonance imaging (MRI) [17,18]. Furthermore, different MNPs have FDA approval for clinical use e.g., Combidex^®^ (Advanced Magnetic Inc., Cambridge, USA, MRI contrast agent for differentiation of metastatic and non-metastatic lymph nodes), Endorem^®^ (Amag Pharmaceutical Inc., Cambridge, USA, MRI contrast agent for diagnosis of liver tumors), Resovist^®^ (Bayer Schering Pharma AG, Berlin, Germany, MRI contrast agent for diagnosis of liver metastases and colon cancer), Feraheme^®^ (Amag Pharmaceutical Inc., Cambridge, USA, indicated for the treatment of iron deficiency anemia in adult patients with chronic kidney disease), *etc*. Another challenging application of MNPs is magnetic hyperthermia [19]. The term hyperthermia refers to the temperature increase of a targeted body tissue/organ, a protocol commonly used in clinic for cancer cell sensitization before radio- or chemotherapy. The magnetic hyperthermia selectively heats up tissues by applying an alternating magnetic field (AMF) to the target tissues previously loaded with MNPs, which act as magnetic mediators for heat generation. This treatment which is highly selective, with minimal damage to surrounding tissue, is currently under clinical trial (Magforce by MagForce Nanotechnologies AG, Berlin, Germany, Food and Drug Administration (FDA, USA) approval for cancer treatment in 2010) for the treatment of patients with recurrent glioblastoma [20,21].

Although the MNPs have not yet been tested on humans for ocular applications, recently their usage in eye disease therapies has been proposed (U.S. Patent 20130225906) [22] and there are evidences from studies in rats that the iron oxide MNPs are non-toxic to the ocular structures [23].

In the present study, we have investigated the ability of MNPs to target RPE by IVT injection, using wild type *Xenopus laevis* as model system. *Xenopus* is one of the principal animal models used in developmental biology since 1950 and much of our knowledge regarding the mechanisms of early vertebrate development are derived from these studies. *Xenopus* offers favorable features [24], such as external development, large supply of embryos with each fecundation, a very short early development time (three days to reach tadpole) and close homology with human genes. A remarkable similarity in the molecular signaling processes, cellular structure, anatomy, and physiology of eye has been observed among *Xenopus* and other high-order vertebrates, including humans [24]. Their relatively large size (from 1–1.2 mm (zygote) to 1 cm (120 h post fertilization)) enables an easy manipulation and IVT microinjection. Finally, the use of this model generates minor ethical issue compared to mammals. Stages from fecundation to the time at which they become capable of independent feeding (stage 45) do not require a license as these embryos are considered to not be sufficiently sentient, or experience nociceptive sensations when subjected to experimental procedures.

In the following experiments, the particles were injected intravitreally, *i.e*., in the anterior part of the eye and, specifically, in a region behind the lens surrounded by the vitreous humor. Biodistribution and localization studies were performed and the effect of the particle size and charge was also assessed.

## 2. Results and Discussion

### 2.1. MNP Biodistribution

**Figure 1.**
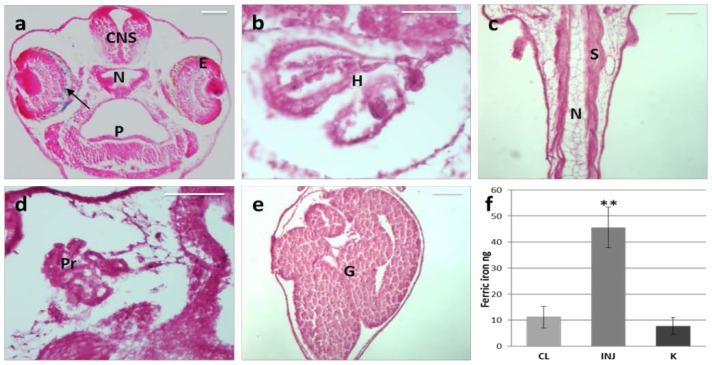
Distribution of MNPs 24 h after IVT injection in the left eye of *Xenopus embryos*. (**a**–**e**) Prussian Blue staining on paraffin section. The arrow points to blue labeled MNPs. CNS, central nervous system; E, eye; N, notochord; P, pharynx; H, heart; S, somites; Pr, pronephros; G, gut; (**f**) Iron content assay on explanted eyes. CL, contralateral eye (non-injected); INJ, injected eyes; K, wild type eyes. Scale bar, 50 μm. *n* = 3, ** *p* < 0.001, *t*-test.

We used commercial fluorescent MNPs with a negative surface charge and a hydrodynamic size of 252 nm (Table 1) to analyze their biodistribution. We injected the MNPs into the left eye of *Xenopus* larvae (stage 37). Twenty-four hours after the injection, we observed, by Prussian blue staining of paraffin sections, MNPs specifically retained in the ocular tissues without any diffusion to the other tissues, including the contralateral eye (Figure 1a–e). A strictly targeted localization of the nanocarrier is one of the crucial objectives of an efficient drug delivery to avoid systemic side effects or collateral effects on normal adjacent tissues. Quantitative analysis of the iron content by thiocyanate colorimetric assay was used to confirm that all the MNPs were retained in the injected eye. For this analysis, stage 37 embryos were subjected to IVT injection of 70 ng of MNPs, corresponding to 52.7 ng of ferric iron, in the left side. Injected and contralateral eyes were explanted 24 h after injection and compared to eyes of wild type embryos. Notably, the average ferric iron content in the injected eyes was significantly higher than in the control eyes (*p* = 0.008, Figure 1f). Specifically, we recorded 45.6 ± 7.7 ng of ferric iron in the treated eyes, *i.e*., retention of 71.79% (normalized with the respect of control K) of the total injected amount. The small loss of ferric iron is compatible with a little reflux from the injection hole. These results, together with the histochemical observations, demonstrate that the MNPs are retained exclusively inside the injected eye.

**Figure 2.**
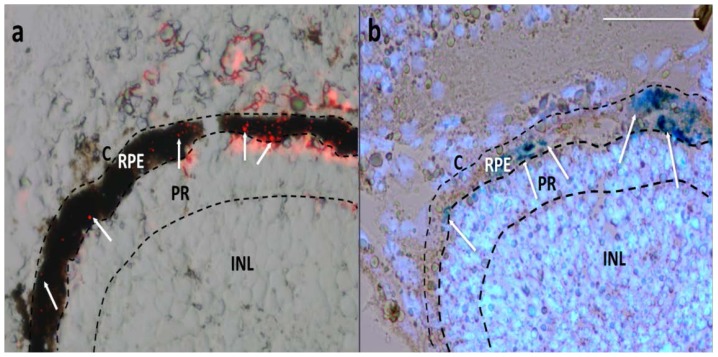
Cryostat sections of *Xenopus* embryos 1 day after injection. (**a**) Merged image of bright field (showing the pigmented RPE) and red fluorescence field (showing MNPs as red spots); (**b**) Image from a bleached section merging the bright field (showing MNPs as dark blue spots by Prussian Blue staining) and fluorescence field (showing fluorescent blue nuclei by Hoechst staining). White arrows point some MNPs. C, choroid; RPE, retinal pigmented epithelium; PR, photoreceptors; INL, inner nuclear cell layer. Scale bar, 50 μm.

**Figure 3.**
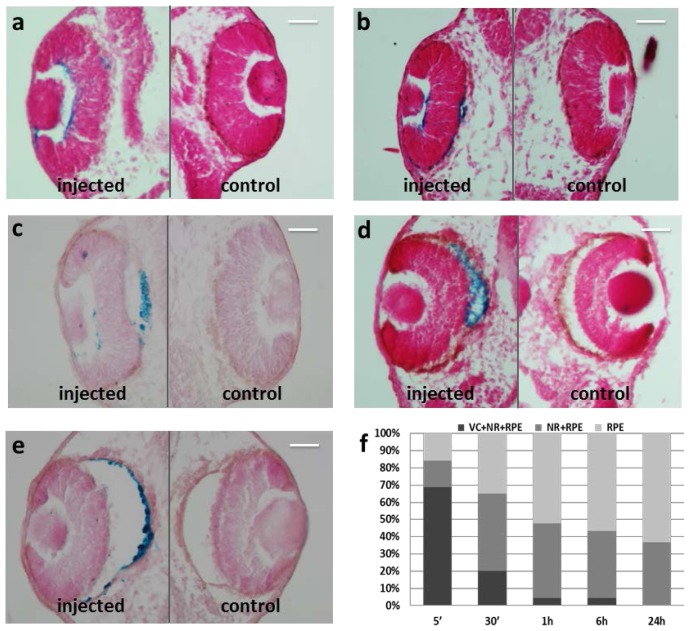
Prussian Blue staining on paraffin section of *Xenopus* embryos. (**a**) 5 min after injection; (**b**) 30 min after injection; (**c**) 1 h after injection; (**d**) 6 h after injection; (**e**) 24 h after injection; (**f**) Graphical representation of MNP localization in eye regions of the embryo population. VC, vitreous chamber; NR, neural retina; RPE, retinal pigmented epithelium. *n* = 45 each time point. MNPs are blue labeled. Scale bar, 50 μm.

It is important to note that we did not observe any toxic effects on the ocular structures caused by MNPs. This observation is in agreement with a recent report by Raju *et al*., confirming absence of toxicity signs in rat ocular tissues, following injection of magnetic micro- and nanoparticles [23]. Although *Xenopus* model has differences with respect to higher species, it constitutes a very sensitive tool for the assessment of toxicity. Highly orchestrated events are required for normal development; therefore any perturbations by a toxic agent will disrupt orderly embryogenesis within 24 h of injection, resulting in abnormal development, manifested as morphological malformations, behavioral changes and even death of the embryo. In this study, no death or embryonic malformations were observed in MNP treated embryos and the injected eye exhibited completely normal development (Figures 1–3).

### 2.2. Particles Injected Intraocularly Localize behind the Neural Retina

Even when the particles were injected in the anterior part of the eye, around the lens in the vitreous chamber, they localized preferentially in the posterior segment, in a region corresponding to the choroid/RPE. The choroid and RPE are thin layers located between the sclera and the neural retina. The choroid is one of the two vascular layers of the eye and lies closest to the sclera while RPE is a single layer of pigmented cuboidal epithelial cells adjacent to the neural retina. In order to define precisely the MNP localization after one day from the injection, we studied the fluorescence of MNPs on cryostat sections without pigment bleaching, to highlight RPE (Figure 2a). In this way, we established the precise localization of the MNPs, as red spots, in RPE. MNPs show a patchy distribution inside RPE, likely due to dipole-dipole interactions which occur when they cross biological barriers [25]. Specifically, when nanoparticles cross retinal structures, they concentrate, interact magnetically with each other, and cluster. A similar mechanism occurs when particles cross cellular membranes. As a consequence, we found that different RPE cells engulf different amounts of MNP clusters. The moderate red background is most likely due to a partially degradation of the MNP linked fluorophore, as confirmed by the histological staining (Prussian Blue, Sigma-Aldrich, St. Louis, MO, USA) of particles which were found to co-localize only with RPE nuclei layer (Figure 2b).

It is conceivable that MNPs injected intravitreally (around the lens in the superficial eye region) migrate through the eye structures until they reach their specific localization in RPE, as previously reported for PLA nanoparticles [8] and HSA nanoparticles [9]. In the study of Bourges *et al*. [8], PLA NPs IVT injected in rat eyes were found to diffuse from the vitreous cavity in all directions. During the first month after the IVT injection, some NPs were observed to be attached to the posterior capsule of the lens, in the iris and ciliary body, and within the vitreous. Four months after the injection, the detected particles were absent from the vitreous cavity, but localized within the intraocular tissues and concentrated in RPE cell layer. Similar results were reported by Kim *et al*. [9] who demonstrated that HSA-NPs, IVT injected in rat eyes, penetrated the whole retina, localized inside RPE but were not able to cross RPE. Different results were reported by Raju *et al*. [23] in studies on nano- and micromagnetic particles localization after IVT injection in the rat eye. In this study, 4 μm particle clusters were detected in histologic sections of the retina where they were still detectable after five months. In contrast, 50 nm MNPs were never detected in histologic sections, but were visible by immunofluorescence in the ganglion cell layer. However, it is not possible to compare the results of this study with those of the present study as the particles used by Raju *et al*. [23] consisted of polymer coated iron oxide conjugated to goat anti-mouse IgG. Moreover, details of surface charge and hydrodynamic radius after functionalization were not provided, which is important as it is well documented from the reported literature that surface properties of nanomaterials mediate cellular interaction and drive their localization and fate *in vivo* [6].

In order to characterize the kinetics of the migration process, we monitored the localization of MNPs at different time points starting from 5 min to 24 h after injection. Just 5 min after injection, the particles started to spread out from the vitreous chamber (VC), adhering to the neural retina (NR) and only few were in RPE (Figure 3a). Within 30 min of injection, the fraction of MNPs observed within the vitreous decreased because of particle migration toward the NR and RPE (Figure 3b) and this decrease became more evident 1 h after injection (Figure 3c). Few to no MNPs were detected at 6 h in the VC and in the NR, while the amount of particles which had localized in RPE increased (Figure 3d). Approximately 24 h after injection, the majority of MNPs had accumulated preferentially within RPE cells, with only a negligible amount of particles being still present in NR (Figure 3e). These results indicate that the MNPs progressively migrate at RPE from the vitreous chamber (Figure 3b–d) and that the migration process is completed within 24 h (Figure 3e,f). This localization dynamics matches that of PLA nanoparticles observed in adult rat [8], although MNPs in *Xenopus* embryos reach RPE within 5 min in contrast to 6 h in rats. This could be explained by the small volume of vitreous humour in embryos eyes compared to adult eyes, enabling higher migration rates, or to intrinsic different migration capability of PLA and MNPs.

**Figure 4.**
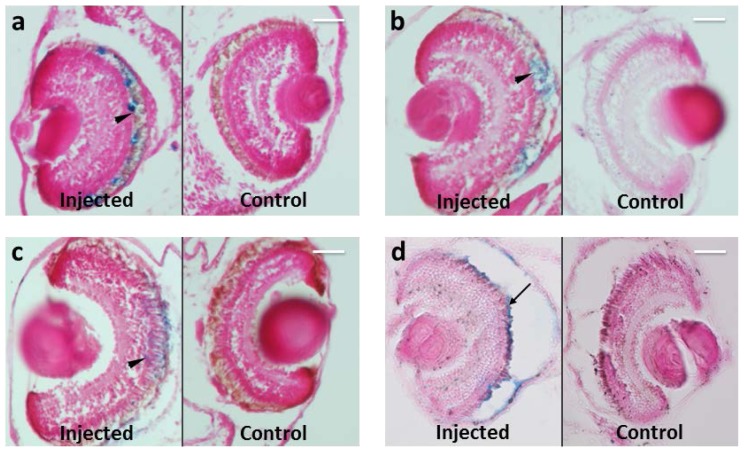
Prussian Blue staining on paraffin section of *Xenopus* embryos at different time points from MNPs injection. MNPs are blue labeled. *n* = 45 each time point. (**a**) Three days after injection; (**b**) five days after injection; (**c**) 10 days after injection; (**d**) 20 days after injection. Arrowheads point to MNP localization in interdigitated RPE microvilli with the outer segment of photoreceptors; arrow points to MNPs derived from RPE rupture, during manipulation (details in the material and methods section). Scale bar, 50 μm.

Another crucial factor for efficient drug delivery is permanence at the intended target site. For this reason, we monitored the retention of MNPs in embryos for periods up to 20 days. Permanence at the intended site is very important in effective drug delivery systems based on MNPs because these particles are, in principle, biodegradable. Indeed, *in vivo*, the body is able to degrade these particles into their elements which then enter in the normal iron metabolic pathways [26,27]. In this aspect of the study, we demonstrated that MNPs localized in RPE at all-time points studied: 3, 5, 10 and 20 days after injection (Figure 4). The MNP localization follows RPE during its development, included in the later stages where RPE microvilli interdigit with the outer segment of photoreceptors [28] (arrowheads in Figure 4a–c). Since the outer segments of photoreceptor are strongly interdigitated with RPE microvilli, it is possible that during the mechanical rupture part of RPE, in particular the microvilli, and thus associated MNPs, remain adherent to the photoreceptor layer (Figure 4d, arrow). These considerations enable prediction on the dynamics of MNP trafficking in the eye. Specifically, the exclusive presence of MNPs in RPE at the long time points, suggests that MNP presumably are degraded in loco by iron dissolution, excluding other exit mechanisms, such as passage through the hyaloid into the anterior chamber, exit through the trabecular meshwork or choroid layer and transport in lymphatic or systemic circulations.

### 2.3. MNP Localization Is not Driven by Superficial Charge and Size

**Figure 5.**
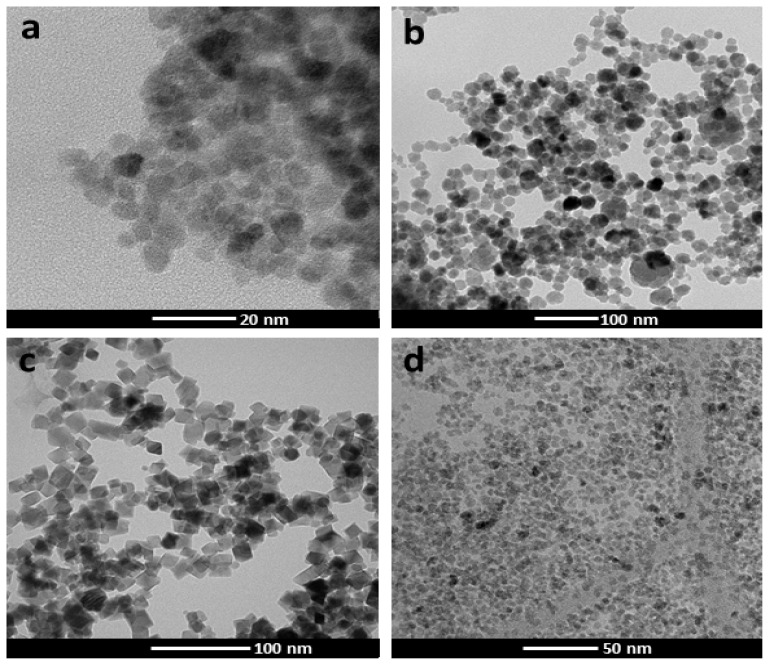
(**a**) TEM images of MNP; (**b**) TEM images of MNP^−^; (**c**) TEM images of MNP^+^; (**d**) TEM images of MNP^s^.

**Figure 6.**
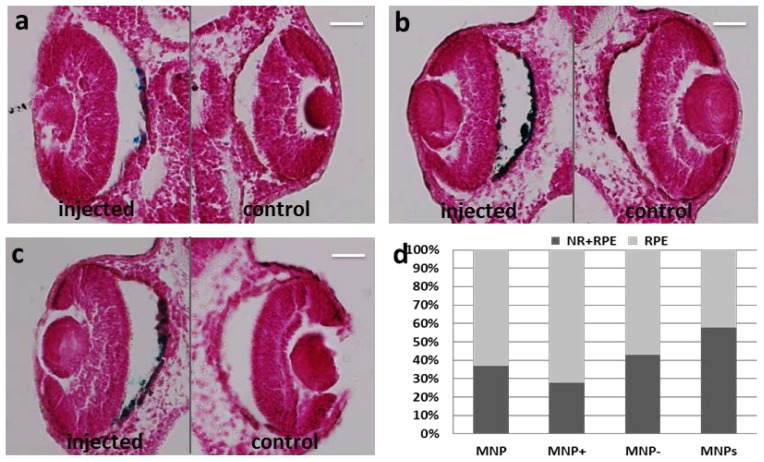
Prussian Blue staining on paraffin section of *Xenopus* embryos one day after injection. Particles are blue labeled. *n* = 45 each group. (**a**) Left eye injected with MNP^−^; (**b**) Left eye injected with MNP^+^; (**c**) Left eye injected with MNP^s^; (**d**) Graphical representation of MNP, MNP^+^, MNP^−^ and MNP^s^ localization in eye regions of the embryo population. VC, vitreous chamber; NR, neural retina; RPE, retinal pigmented epithelium. Scale bar, 50 μm.

**Figure 7.**
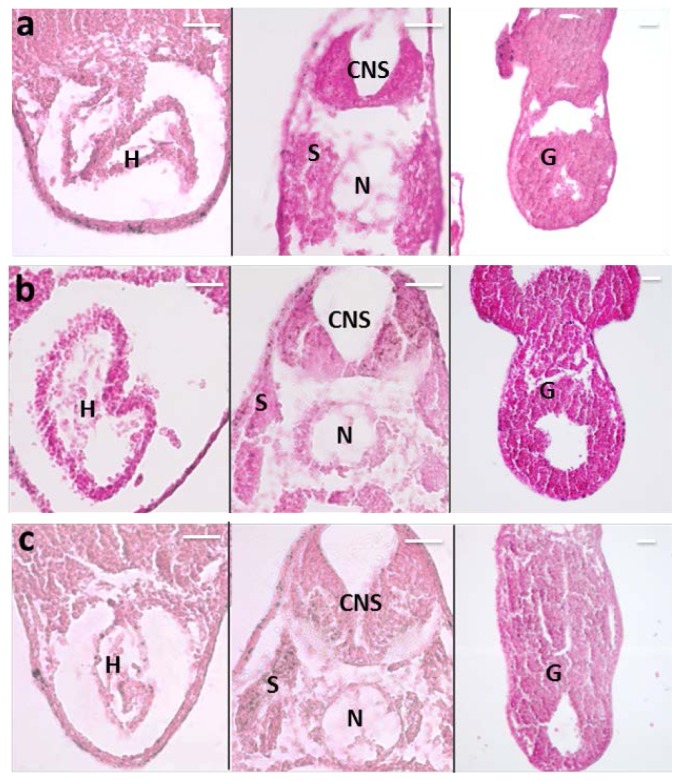
Prussian Blue staining on paraffin section of *Xenopus* embryos one day after injection. Particles are blue labeled. *n* =45 each group. (**a**) Left eye injected with MNP^−^; (**b**) Left eye injected with MNP^+^; (**c**) Left eye injected with MNP^s^. H, heart; CNS, central nervous system; S, somites; N, notochord; G, gut. Scale bar, 50 μm.

It is known that nanoparticle size and surface charge influence the movement of nanoparticle-based ocular drug delivery systems [6]. Because of the high viscosity of vitreous humor (two to four times higher than pure water), different sized nanoparticles, injected into the vitreous cavity of unilateral eyes of pigmented rabbits, diffuse differently in the vitreous and in ocular tissues, with a half-life elimination from the vitreous cavity, which closely correlated with the particle diameter [7]. Specifically, 2 μm diameter particles diffuse in the vitreous cavity to the trabecular meshwork, while smaller nanoparticles (<200 nm) were also observed in the retina and other tissues. One study demonstrated the importance of charge on the intravitreal movement of nanoparticles [9]. It has been suggested that hyaluronic acid, a negatively charged glycosaminoglycan abundant in vitreous, may interact with cationic complexes. Peeters *et al*. [29] showed that cationic liposome complexes clearly aggregated in the vitreous while the binding of the liposome to the biopolymers in the vitreous is reduced by decreasing the zeta potential to become anionic. In another study, IVT injected anionic HSA-NPs diffused freely in the posterior direction from the vitreous to the retina, while IVT injected cationic HSA-NPs were bound to and aggregated in the vitreous [9]. Based on these reported studies, we investigated the effect of size and charge on MNP movement by comparing the localization of our MNPs (250 nm, −17 mV) with the localization of particles of similar size but more negatively charged (polyacrylic acid-coated MNPs, hereafter labeled as MNP^−^) (data unshown), or positively charged (polyethyleneimine-coated MNPs, hereafter labeled as MNP^+^) [30], or particles with neutral charge but small size (Feraheme^®^ MNPs; Cambridge, MA, USA, hereafter labeled as MNP^s^). The morphology of the particles used in this study is shown in Figure 5, and Table 1 summarizes the particle features, in terms of hydrodynamic diameter and surface potential. Surprisingly, the results were the same with all kinds of MNPs, *i.e*., they localized in RPE one day after injection (Figure 6). No particles where detected in VC and most of them were found in RPE with only a small fraction in NR for all kinds of MNPs analyzed (Figure 6d). Moreover, also with all MNP^−^, MNP^+^ and MNP^s^ there was no particles diffusion to extra-ocular tissues (Figure 7).

For the first time, we demonstrated that charge surface, beyond the size, does not influence the localization of nanoparticles in RPE. We speculate that MNPs, with different charge and size, can diffuse in the vitreous, infiltrate among retinal neurons without cells engulfing until they reach RPE. These cells have a strong phagocytic activity, required for maintaining constant renewal process of the photoreceptor outer segments [31]. Consistent with this, it has been previously demonstrated that RPE cells are able to ingest microspheres *in vitro* [32] and *in vivo* [33] after subretinal injection.

The peculiar properties of MNPs could provide a possible explanation of the mechanism of particle localization in RPE. First, their nanometric size could allow MNPs to diffuse across the porosity of the vitreous humor which is a highly-hydrated network of protein fibrils and charged polysaccharide chains. Second, dipole-dipole interactions could occur during the particles penetration across the sensory retina, leading to the formation of bigger aggregates. Finally, the formation of such clusters could facilitate phagocytosis by RPE cells which can easily internalise particles of micrometric size [32]. According to this, MNPs could be proposed as carriers for sustained intracellular delivery of drugs, especially those characterised by low retention in RPE cells due to drug efflux mechanisms.

### 2.4. MNP RPE/Choroid Localization Is not Species-Specific

**Figure 8.**
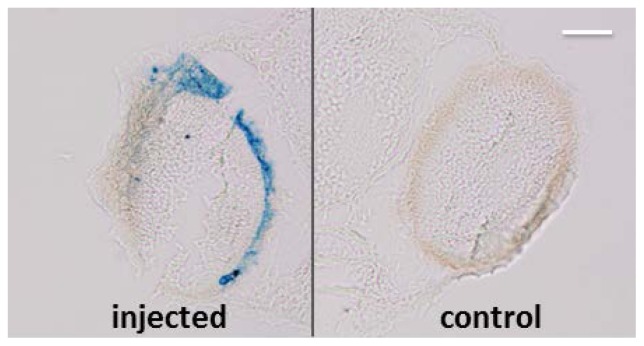
Prussian Blue staining (without pararosaniline counterstaining) on paraffin section of zebrafish embryos one day from injection. MNPs are blue labeled. Scale bar, 50 μm. *n* = 45.

In order to understand if the capability of MNPs to localize in RPE is species specific, we injected 3.5 ng of particles in the left eye of zebrafish embryos at 48 h post fertilization. We found that one day after injection the MNPs localize specifically in RPE in zebrafish as well as in *Xenopus* (Figure 8). This datum suggests that the localization of MNPs in RPE is not species-specific. Further experiments will be devoted to demonstrate RPE-specific localization of MNPs also in mammalians, as already demonstrated with other NPs [8,9] but not yet with MNPs [23].

## 3. Experimental Section

### 3.1. Nanoparticles

MNP used in this work are nano-screenMAGARA supplied by Chemicell, The vial, nano-screenMAGARA, as provided by the supplier, contains an aqueous dispersion of magnetic fluorescent nanoparticles 25 mg/mL. The particles have a magnetic core of magnetite and a polysaccharide matrix of glucuronic acid, a derivate of glucose. The magnetite core is overlaid with a red fluorescence dye (excitation: 578 nm; emission: 613 nm). MNP^s^ are Feraheme (ferumxytol) which are magnetite nanoparticles coated with polyglucose sorbitol carboxymethylether, supplied by AMAG Pharmaceuticals (Cambridge, MA, USA) and approved by FDA (USA) to treat iron deficiency anemia in adult patients with chronic kidney disease. Each mL of the sterile colloidal solution of Feraheme (AMAG Pharmaceuticals, Cambridge, MA, USA) injection contains 30 mg of elemental iron and 44 mg of mannitol. The formulation is isotonic with an osmolality of 270–330 mOsm/kg.

The polyacrylic acid-coated Fe_3_O_4_ nanoparticles (labeled MNP^−^) were synthesized through a modified oxidative hydrolysis method, based on the precipitation of FeSO_4_ in basic media (NaOH) with a mild oxidant. Reactants were dissolved in distilled water and bubbled with N_2_, then the polyacrylic acid (PAA) was added dropwise under constant stirring. After complete reaction, the black precipitate was held at 90 °C for 24 h under N_2_ and finally cooled to room temperature with an ice bath.

A similar method was used for the polyethyleneimine-coated Fe_3_O_4_ nanoparticles (MNP^+^), which has been described in detail elsewhere [30].

#### 3.1.1. Transmission Electron Microscopy (TEM)

MNP distribution and morphology were analyzed by transmission electron microscopy (TEM) using a FEI Tecnai T20 microscope (Hiilsboro, OR, USA) and operating at 200 keV. TEM samples were prepared by placing one drop of a dilute suspension of nanoparticles in water on a carbon-coated copper grid and allowing the solvent to evaporate at room temperature.

#### 3.1.2. Dynamic Light Scattering (DLS) and Zeta Potential

DLS and zeta potential were evaluated in water at room temperature using 90 Plus Particle Size Analyzer and Zeta Potential Analyzer (Brookhaven Instrument Corp., Holtsville, NY, USA). Zeta potential was measured at pH = 7.5 in 0.01 M of KCl.

### 3.2. Embryo Preparation

Animal procedures were performed in strict compliance with protocols approved by Italian Ministry of Public Health and of the local Ethical Committee of University of Pisa (authorization n. 99/2012-A, 19.04.2012), in conformity with the Directive 2010/63/EU. *Xenopus laevis* embryos were generated and staged as described [34,35]. The larvae were reared at 14 °C in MMR solution [36] and anesthetized in 0.05% tricaine. Zebrafish embryos were obtained by natural mating and staged according to [37]. The embryos were reared at 28 °C in E3 medium [37] anesthetized in 0.02% tricaine.

### 3.3. Embryo Microinjections

Ten nanoliters of 3.5 mg/mL of MNP, MNP^+^, MNP^−^ and MNP^s^ were microinjected in anesthetized larvae of *Xenopus laevis* at stages 37 [34] in the left eye for each replicate. After injection, embryos were reared at 14 °C. One nanoliter of 3.5 mg/mL of MNPs was microinjected in anesthetized larvae of zebrafish at 48 hpf (hour post fertilization) in the left eye for each replicate. Each experiment was replicated three times. Each replicate was performed on 15 larvae.

### 3.4. Histochemical Analysis

Five min, 30 min, 1 h, 6 h, 1 day, 3 days, 5 days, 10 days and 20 days after ocular microinjection, *Xenopus* embryos were formalin fixed for 1 h, after which they were embedded in paraffin and sectioned (14 μm). Detachment of the neuroretina from RPE was found on the control and injected eye to the same degree, and was consistent with tissue processing artefacts [38].

One day after ocular microinjection, zebrafish embryos were fixed in 4% paraformaldehyde for 2 h, after which they were embedded in paraffin and sectioned (10 μm). For cryostat sections the embryos were fixed in 4% paraformaldehyde in PBS for 2 h and cryoprotected with 20% sucrose in PBS for 2 h, then they were embedded in OCT cryostat embedding medium Tissue Tek^®^ (Sakura, Finetek, Torrance, CA, USA) and criosectioned (12 μm). Nuclei staining was performed using hoechst 33342.

The cryo- and paraffin sections were stained by Prussian Blue according to the manufacturer’s instructions (Sigma-Aldrich, St. Louis, MO, USA) after a treatment of pigment bleaching in 50% formamide-1% hydrogen peroxide in presence of cold light.

### 3.5. Iron Content Assay

Forty-eight hours from injection, left (injected) and right (control) eyes were explanted from 30 sacrificed tadpoles and both eyes from 15 sibling wild type tadpoles were treated similarly. Each eye type was pooled and iron content was assessed by thiocyanate colorimetry. Briefly, the samples were incubated for 1 h at 60 °C in HCl 1M plus HNO_3_ 65% to reduce MNPs in ferric iron, then sample was water diluted 1:10 and an equal volume of KSCN 1.5 M was added. Absorbance at 478 nm was immediately recorded. Known concentrations of ferric iron were used to obtain the calibration curve.

## 4. Conclusions

In the present study, we found that MNPs are able of fast and specific localization in RPE layer, in an embryo model for the study of vertebrate diseases. The model offers distinct advantages (small transparent embryos, fast development) and can be exploited in disease processes as a first step for therapeutic proof-of-concept studies, replacing or drastically reducing the use of mammals.

In the present study, we demonstrated that in *Xenopus* embryos MNPs localize autonomously and specifically in RPE after IVT injection independently by particle size and surface charge. Moreover, this process seems to be not species-specific as IVR injected MNPs were found to localize in RPE in zebrafish embryos too.

In conclusion, the MNPs have the potential for development as an ocular drug delivery, capable of targeting RPE with targeted sustained controlled drug release. Furthermore, the MNP carrier system would provide a minimal access therapy and MRI tracking for a variety of retinopathies. Moreover, the MNPs could be exploited also for magnetic hyperthermia treatments of ocular iper-proliferative diseases. Additionally, there are other challenging applications which could be explored for the use of MNPs, such as magnetic targeting of RPE in the treatment of retinal detachment by applying external magnetic forces. The versatility of the animal model proposed here opens the way to systematic exploration of these possibilities with easy access to *in vivo* experiments avoiding the use of small animals for initial proof of concept studies.

## Figures and Tables

**Table 1.** Size (hydrodynamic radius), surface potential (Zp).

	MNP	MNP−	MNP^+^	MNP^s^
Hydrodynamic Radius	252 nm	158 nm	95 nm	17 nm
Zp (@water)	−17.22 mV	−28 mV	+30 mV	0.4 mV

^−^: strongly negative charge; ^+^: positively charge; ^s^: small size.
